# Polyvinyl Alcohol–Citric Acid: A New Material for Green and Efficient Removal of Cationic Dye Wastewater

**DOI:** 10.3390/polym15224341

**Published:** 2023-11-07

**Authors:** Ye He, Yangyang Zheng, Xia Liu, Chang Liu, Huacheng Zhang, Jie Han

**Affiliations:** 1 School of Chemical Engineering and Technology, Xi’an Jiaotong University, Xi’an 710049, China; 2 Shandong Nonmetallic Materials Institute, Jinan 250031, China; 3 Key Laboratory of Advanced Energy Materials Chemistry (Ministry of Education), College of Chemistry, Nankai University, Tianjin 300071, China

**Keywords:** polyvinyl alcohol, citric acid, polymer, methylene blue, adsorption mechanism

## Abstract

The citric acid (CA) cross-linked polyvinyl alcohol (PVA) adsorbent, PVA–CA, was efficiently synthesized and its application to the removal of dyes in water, particularly the cationic dye, methylene blue (MB), was thoroughly investigated. The morphologies and physiochemical characteristics of PVA–CA were fully characterized by SEM, FT-IR, XRD, TGA, BET, and XPS. The effects of contact time, adsorbent dosage, MB concentration, solution pH, and temperature on the adsorption performance were compared using controllable methods. The maximum adsorption capacity of PVA–CA was 709.86 mg g^−1^ and the removal rate remained high through several adsorption–desorption cycles, demonstrating that such a composite absorbent has a good adsorption performance and recoverability. Further analysis by the density functional theory (DFT) showed that van der Waals interactions, electrostatic interactions and hydrogen bonding interactions between PVA–CA and MB played significant roles in the adsorption mechanism.

## 1. Introduction

As a key type of pollutant in water worldwide [1,2,3], dyes from daily life and industrialization cause a crisis for human beings’ survival. Currently, adsorption is a commonly used, efficient, environmentally friendly, and sustainable treatment for dyes [4,5,6]. In adsorption research, methylene blue (MB) as a basic dye is one of the most general dye molecules used for practical experiments [7,8,9]. 

Polyvinyl alcohol (PVA) as a polyhydroxy substance for cross-linking is cheap and easily available, green and degradable, and does not bring secondary pollution to the environment. It is now widely used in food packaging, bioengineering, and wastewater treatment [10,11,12]. Liang et al. [13] prepared water-insoluble supramolecular polymeric adsorbents using PVA as the main chain in combination with CA and β-CD for the treatment of naphthenic-acid-containing oil sand process wastewater. Yu et al. [14] prepared ion-imprinted composite hydrogel materials with PVA as the main non-toxic component and CA as the cross-linker to enhance the stability of ion-imprinted pores. But PVA itself has too good solubility in water and limitations in adsorbing dye molecules. Thus, PVA often needs to be further modified to improve its water-resistance and adsorption performances. One of the most general methods of PVA modification is esterification crosslinking [15,16,17,18]. For example, citric acid (CA) [19], as a green cross-linking agent for polycarboxylic acids, is often used to modify PVA for forming ester bonds with the hydroxyl groups on PVA and fabricating functional materials with diverse water solubility. Particularly, the extra carboxyl groups on CA can have strong hydrogen bonding towards the unreacted hydroxyl groups on PVA, further improving the mechanical properties and thermal stability of the thus obtained polymeric material [20,21]. As both PVA and CA are biodegradable, the polymers prepared by grafting CA onto the PVA backbone are also biodegradable and will not cause secondary pollution to the environment after use [18]. Interestingly, they are often used to prepare polymeric materials by cross-linking with other substances, and have a wide range of applications. For example, membranes based on esterification between CA and PVA have been used in the field of food, fruit, and vegetable preservation [22,23,24,25,26,27,28]. However, their applications in wastewater treatment are very limited [16,29].

PVA is a kind of polyhydroxy substance with good solubility in water. CA, as a kind of green crosslinking agent of polycarboxylic acid, is often used to modify PVA, which can esterify with the hydroxyl group on PVA to prepare a water-insoluble polymer adsorbent. Here, the PVA–CA composite adsorbent was efficiently prepared by using the esterification reaction under the optimum preparation conditions. The effects of the mass ratio between CA and PVA, reaction temperature and reaction time on the adsorption capacity of PVA–CA were thoroughly investigated by single-factor and orthogonal experiments. The materials were fully characterized and analyzed by using SEM-EDS, FT-IR, XRD and TGA. The application of PVA–CA to the removal of cationic dyes MB from the water was also systematically investigated. The effects of contact time, adsorbent dosage, initial concentration of the solution, solution pH, temperature, and the number of cycles were investigated on the adsorption performance of PVA–CA. The adsorption behavior of PVA–CA was analyzed by using an adsorption kinetic model, an adsorption isotherm model, and an adsorption thermodynamic model. In addition, the mechanism of the adsorption process was revealed by FT-IR analysis and DFT calculations. Finally, the adsorption capacity of PVA–CA for different types of dyes was also evaluated using normal experiments.

## 2. Materials and Methods

### 2.1. Materials

The PVA (Mw-20500), CA (>99.5%), potassium dihydrogen phosphate (KH_2_PO_4_, >99.95%), and MB (>98%) were all purchased from Macklin (Shanghai, China) without further purification. Ultrapure water was directly made in the lab.

### 2.2. Synthesis of PVA–CA

The polymeric adsorbent was obtained through the esterification of CA with PVA as follows: the PVA solution was prepared initially with ultra-pure water, and then mixed well with CA and KH_2_PO_4_. The mixed solution was stirred at room temperature until the raw material was completely dissolved, and the as-formed solution was sonicated to eliminate the foam. Then, the solution reacted for 3 h at 130 °C in a blast dryer to obtain a yellow-brown solid product. The resulting solid product was removed and cooled to room temperature. Unreacted raw materials and impurities were removed via soak and wash with ultrapure water. Finally, the solid product was placed in a vacuum oven and dried to a constant weight at 50 °C for 24 h to produce the polymeric adsorbent—PVA–CA. The specific preparation process is shown in Figure S1. The results of the single-factor experiments are shown in Figures S2–S5. The results of the orthogonal experiments are shown in Tables S1 and S2.

### 2.3. Characterization

The morphologies of the PVA–CA adsorbent were examined by scanning electron microscopy (SEM) (MAIA3 LMH, Warrendale, PA, USA). The surface chemistry of the material was analyzed by Fourier transform infrared spectroscopy (FT-IR) in the range of 4000–400 cm^−1^ (Nicolet iS50) at room temperature. Thermogravimetric analysis (TGA) was carried out by NETZSCH STA449F5 in the range of 30–500 °C at a heating rate of 10 °C min^−1^ in a nitrogen atmosphere to study the stability of the material. The X-ray diffraction (XRD) analysis was carried out using Shimadzu XRD-6100 with Cu Kα radiation (Condition: λ = 0.15406 nm, 100 mA, 40 kV, scan rate 6° min^−1^, and range of 2θ = 10°~80°). The specific surface areas of these adsorbents were estimated using the nitrogen adsorption–desorption method (TriStar II 3020 3.02) using the Brunauer–Emmett–Teller (BET) equation. The energy-dispersive X-ray spectrometer (EDS, Aztec X-max N-50 mm^2^, Concord, MA, USA) was employed to investigate the elemental distribution of these adsorbents. X-ray photoelectron spectroscopy (XPS) data were obtained by the Thermo Fisher ESCALAB Xi^+^ photoelectron (Waltham, MA, USA) using Al Kα excitation. A UV–VIS absorption spectrometer (TU-1901 Beijing Purkinje GENERAL Instrument Co., Ltd., Beijing, China) was used to determine the concentrations of MB left in supernatant solutions, referring to the standard curve of MB at the maximum wavelength (664 nm).

### 2.4. Adsorption Experiment

#### 2.4.1. Determination of Methylene Blue Solution Standard Curve

A series of MB standard solutions (2.0, 4.0, 6.0, 8.0, 10.0, 15.0, 20.0, and 25.0 mg L^−1^) were prepared in the concentration range of 2.0 to 25.0 mg L^−1^. The absorbance of the solutions was measured at a wavelength of 665 nm [30] and the average of three measurements were taken for each concentration. The standard curve of MB was then fitted with the concentration of MB (C, mg L^−1^) as the horizontal ordinate and the absorbance (Abs) as the vertical ordinate, as shown in Figure S6. The standard curves of Congo Red (CR), Tartrazine (TZ), Malachite green (MG), and Rhodamine B (RB) were plotted in the same way, as shown in Figure S7.

#### 2.4.2. Adsorption Performance Evaluation

A 100 mL conical flask was filled with 20 mL of the desired concentration of MB at an initial pH of 7.0. A certain amount of adsorbent was then added and the conical flask was then placed into a gas bath thermostatic shaker and shaken at a set temperature at an oscillation rate of 130 r min^−1^ until the adsorption reached equilibrium. The absorbance of the adsorbed MB was then measured using a UV spectrophotometer. The residual concentration of MB was determined from the standard curve of MB in Section 2.4.1. The adsorption capacity q_e_ (mg g^−1^) and removal rate R% of MB by the adsorbent can be calculated from Equations (1) and (2).

(1) $$q_{e}=\frac{(C_{0}-C_{e})V}{m}$$

(2) $$R\%=\frac{C_{0}-C_{e}}{C_{0}}\times 100\%$$

where $C_{0}$ (mg L^−1^) and $C_{e}$ (mg L^−1^) are the concentration of MB in the initial and at equilibrium time, respectively, and V (L) and m (g) are the volume of the MB and the weight of the adsorbent, respectively.

#### 2.4.3. Single-Factor Experiments

The effects of CA to PVA mass ratio, solution pH, reaction temperature, reaction time, and catalyst amount on the adsorption performance of PVA–CA were investigated separately. The effect of the mass ratio of CA to PVA on the adsorption performance of PVA–CA was investigated at a controlled reaction temperature of 130 °C and a reaction time of 3 h using KH_2_PO_4_ as the catalyst. The effect of reaction temperature on the adsorption performance of PVA–CA was investigated at a mass of CA to PVA of 3:1 and a reaction time of 3 h. The effect of reaction time on the adsorption performance of PVA–CA was investigated at a CA to PVA mass of 3:1 and a reaction temperature of 130 °C. The effect of the amount of KH_2_PO_4_ on the adsorption performance of PVA–CA was investigated at a reaction temperature of 130 °C and a reaction time of 3 h at a mass of 3:1 between CA and PVA.

#### 2.4.4. Recyclability of PVA–CA

Recyclability is one of the most important factors when evaluating the performance of an adsorbent. It was investigated by preparing a sufficient amount of 100 mg L^−1^ MB with an initial pH of 7.0, adding 20 mL of the MB to a 50 mL conical flask, putting 10 mg of PVA–CA adsorbent into the 100 mg L^−1^ MB, capping the conical flask and placing it in a gas bath thermostat shaker. The temperature was set to 30 °C; in turn, the adsorbent was shaken to equilibrium at a rate of 130 r min^−1^, and the absorbance of the adsorbed MB was measured using a UV spectrophotometer. The adsorbent was then filtered out into a 1 mol L^−1^ HCl solution and washed by soaking several times to elute the MB. The adsorbent was then removed and washed clean of the residual HCl with ultrapure water; after that, it was dried to a constant weight and the above steps were repeated. The recyclability of the adsorbent could then be assessed based on the change in the adsorption capacity of the adsorbent after several adsorption–desorption cycles.

### 2.5. Adsorption Kinetics

Adsorption kinetics can describe the time required to complete the adsorption process. It is important for understanding the reaction mechanisms and pathways. Here, two classical adsorption kinetic models were used to analyze the adsorption behavior of CA-based polymeric adsorbents on MB—the quasi-first-order kinetic model (Pseudo-first-order) and the quasi-second-order kinetic model (Pseudo-second-order).

K_1_ (min^−1^) and K_2_ (g mg^−1^ min^−1^) are the adsorption rate constants and pseudo-second-order adsorption rate constants, respectively; q_e_ (mg g^−1^) is the adsorption capacity; and q_t_ (mg g^−1^) is the adsorption capacity at a moment.

### 2.6. Adsorption Isotherm

To investigate the relationship between the initial concentration of adsorbent and the equilibrium adsorption capacity of the adsorbent, the equilibrium adsorption data were fitted using two adsorption isotherm models, i.e., Langmuir (5) and Freundlich (6). The parameters related to the adsorption properties and the maximum adsorption capacity were obtained.

(3) $$\log\left(q_{e}-q_{t}\right)=logq_{e}-\frac{k_{1}t}{2.303}$$

(4) $$\frac{t}{q_{t}}=\frac{1}{K_{2}q_{e}^{2}}+\frac{t}{q_{e}}$$

(5) $$\frac{C_{e}}{q_{e}}=\frac{C_{e}}{q_{m}}+\frac{1}{K_{L}q_{e}}$$

(6) $$\ln\left(q_{e}\right)=lnK_{F}+\frac{1}{n}ln(C_{e})$$

C_e_ (mg L^−1^) is the equilibrium concentration, q_e_ (mg g^−1^) is the equilibrium adsorption capacity, q_m_ is the maximum adsorption capacity, K_F_ and n are constants of the Freundlich equation, and K_L_ is constant for the Langmuir equation.

### 2.7. Adsorption Thermodynamics

The thermodynamics of adsorption were determined using three basic thermodynamic parameters, namely Gibbs free energy change (ΔG^0^, KJ mol^−1^), enthalpy change (ΔH^0^, KJ mol^−1^), and entropy change (ΔS^0^, J mol^−1^ K^−1^). These are used to determine whether the adsorption process is exothermic or heat-absorbing, whether the adsorption process is spontaneous or not, and whether the stoichiometry at the solid–liquid interface is increasing or not. This is important for exploring the mechanism of adsorption [31,32]. The main equations involved are as follows [33].

(7) $$K_{d}=\frac{q_{e}}{C_{e}}$$

(8) $$lnK_{d}=\frac{∆S^{0}}{R}-\frac{∆H^{0}}{RT}$$

(9) $$∆G^{0}=∆H^{0}-T∆S^{0}$$

K_d_ (L g^−1^) is the distribution factor, q_e_ (mg g^−1^) is the equilibrium adsorption capacity, C_e_ (mg L^−1^) is the equilibrium concentration, R (8.314 J mol^−1^ K^−1^) is the general gas constants, and T (K) is the absolute temperature.

### 2.8. Computational Methods

The mechanism of PVA–CA adsorption on MB can be explored by the density general function theory. All DFT calculations during the study were completed in Gaussian16 and ORCA5.0.3 [34,35] software to model the isolated adsorption of PVA-MB and CA-MB. The adsorption energy can be calculated by the following equation:

(10) $$∆E=E_{adsorbent-MB}-E_{adsorbent}-E_{MB}$$

$E_{\mathrm{adsorbent}-\mathrm{MB}}$ is the total energy of the optimized complex of PVA-MB or CA-MB, $E_{\mathrm{adsorbent}}$ is the energy of PVA or CA, and $E_{\mathrm{MB}}$ is the energy of the MB. An independent gradient model based on Hirshfeld partition (IGMH) analysis of wave function files was generated by single point energy calculations with Multiwfn3.8 analysis software and Visual Molecular Dynamics1.9.4 visualization software [36].

## 3. Results and Discussion

### 3.1. Characterization

#### 3.1.1. SEM Analysis

**Figure 1:**
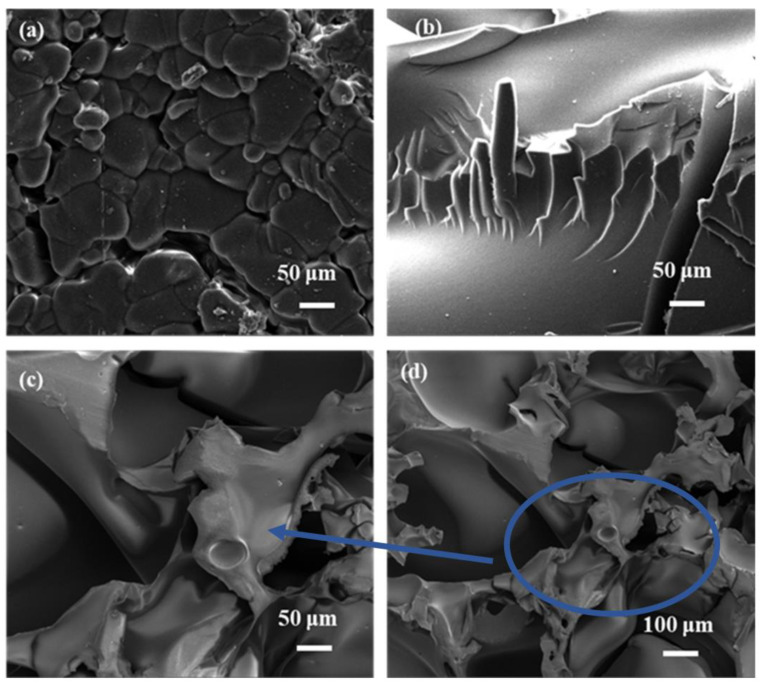
The SEM images of CA (**a**), PVA (**b**), and PVA–CA (**c**,**d**). (**c**) Magnified from an area of (**d**).

After the fabrication of hybrid composites, the morphologies of diverse contents were fully changed. For example, from Figure 1a, the morphology of CA was irregularly granular and closely packed together with some gaps between the particles. In contrast, the PVA in Figure 1b shows the plate structure, which was overall smooth and did not possess a porous structure with some grooves. Interestingly, from Figure 1c,d, the morphology of PVA–CA was quite different from that of CA and PVA, showing a broken flower-like structure with more cracks, folds, and pores, which was beneficial for increasing the specific surface area of the material and provided more adsorption sites towards MB. Thus, the different morphologies of PVA–CA from CA and PVA also confirmed that the esterification between PVA and CA was achieved.

#### 3.1.2. FT-IR Analysis

**Figure 2:**
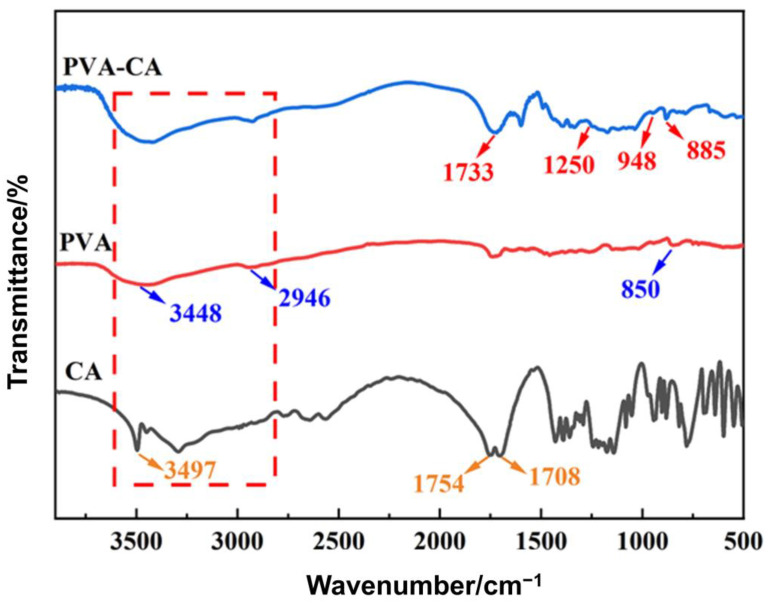
Infrared spectra of CA, PVA, and PVA–CA.

As shown in the FT-IR spectrum of CA (Figure 2), the absorption peak at 3497 cm^−1^ was related to the hydroxyl group on CA, and the absorption peaks at 1754 cm^−1^ and 1708 cm^−1^ followed the symmetrical carboxyl group on CA. In addition, the characteristic absorption peak of PVA appeared at 3448 cm^−1^. This can be attributed to the stretching vibration of the hydroxyl group, the characteristic absorption peak at 2946 cm^−1^ was the symmetric stretching vibration of the main chain methylene group, and the absorption peak at 850 cm^−1^ was the bending vibration of the methylene group. The FT-IR spectrum of PVA–CA showed that the characteristic absorption peak at 1733 cm^−1^ belonged to carbonyl groups [37], and the absorption peak at 1250 cm^−1^ could be attributed to the asymmetric stretching vibration of C–O–C. In addition, the bending vibration absorption peaks of C–O–C at 948 cm^−1^ and 885 cm^−1^ indicated the presence of ester structures. Thus, the esterification between CA and PVA was confirmed, leading to the formation of polymeric architectures.

#### 3.1.3. XRD Analysis

**Figure 3:**
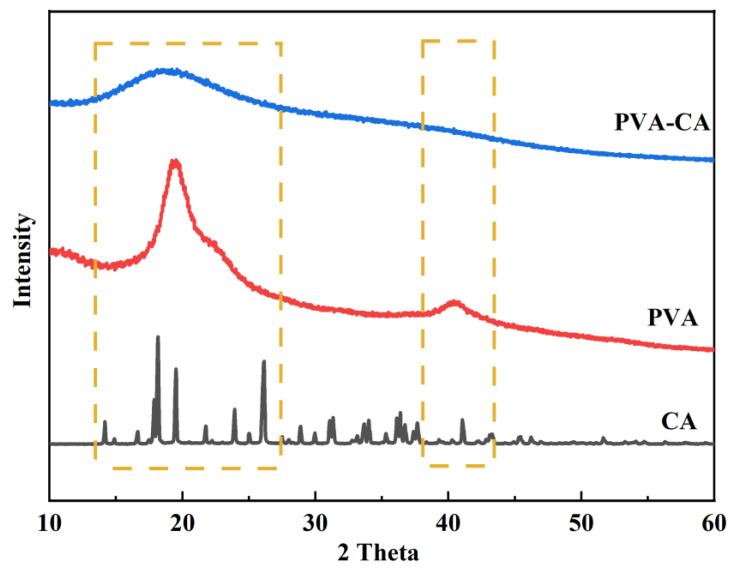
XRD spectra of CA, PVA, and PVA–CA.

The XRD spectra were collected to characterize the crystalline structures of the CA, PVA, and PVA–CA adsorbents. Figure 3 shows many sharp diffraction peaks of CA at 2θ ≈ 14.17°, 18.16°, 19.50°, 26.13°, 36.37°, and 41.04°, indicating its clear crystal structure. The XRD spectrum of PVA showed two broader diffraction peaks at 2θ ≈ 19.50° and 40.40°, indicating that PVA did not have a distinct crystal structure. Different from the cases of CA and PVA, PVA–CA only showed a broader diffraction peak at 2θ ≈ 18.60°, indicating that its structure was amorphous and distinct from that of PVA. Thus, these results indicate that an esterification cross-linking reaction between CA and PVA completely occurred and PVA–CA had quite different structures from its previous pieces [38].

#### 3.1.4. TGA Analysis

**Figure 4:**
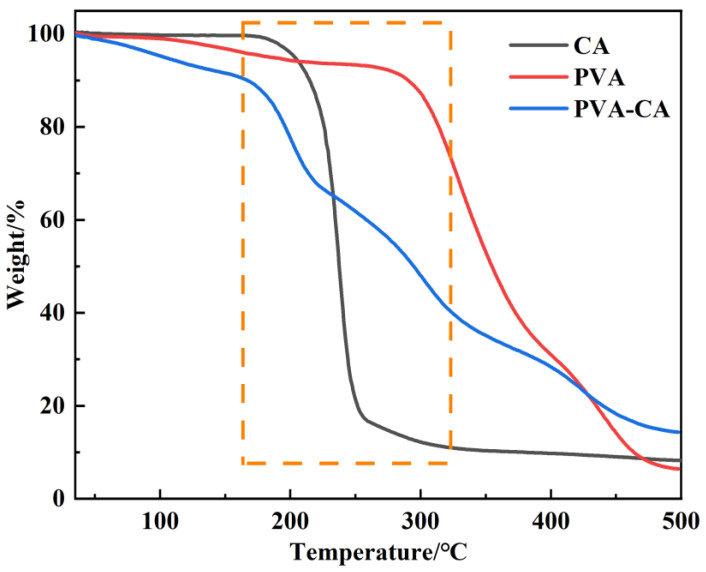
TGA curves of CA, PVA and PVA–CA in N_2_ atmosphere.

Figure 4 shows the mass loss of CA, PVA, and PVA–CA below 100 °C was 0.22, 1.03, and 4.76% respectively, which was related to the evaporation of water in the materials. As shown, the respective decomposition temperatures of CA, PVA, and PVA–CA were 176, 270, and 157 °C, respectively. Between 176 and 300 °C, the mass loss of CA was approximately 90%, between 270–500 °C the mass loss of PVA was approximately 86%, and between 157–500 °C the mass loss of PVA–CA was approximately 76%, which is caused by the decomposition of material structures. Additionally, the residual masses of CA, PVA, and PVA–CA at 500 °C were 8.25%, 6.41%, and 14.3%, respectively. Interestingly, the structure of PVA–CA decomposed a bit earlier than that of CA and PVA, but its residual mass at 500 °C was much higher than that of other cases. This indicates that the thermal stability of the polymer weakened a lot after cross-linking by esterification [39].

### 3.2. Investigation of MB Adsorption Performance

#### 3.2.1. Effect of Dose on Adsorption Performance

**Figure 5:**
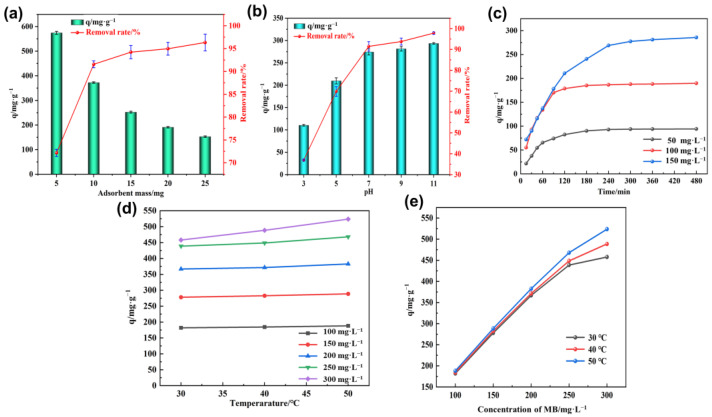
Effect of different factors on the adsorption performance of PVA–CA: dose of adsorbent (**a**), pH (**b**), contact time (**c**), concentration of MB (**d**), and temperature (**e**).

The dose of adsorbent could affect the removal rate and adsorption capacity of the prepared PVA–CA absorbent. Firstly, as shown in Figure 5a, the removal rate of MB by PVA–CA increased with increasing doses. When the dose of PVA–CA was increased from 5 mg to 25 mg, the removal rate increased from 72.17% to 96.33%. At higher doses, the adsorbent could provide more adsorption sites for MB, which led to an increase in removal efficiency. But the removal rate of PVA–CA already reached over 90% at a dosage of 10 mg of adsorbent, and, thereafter, the increase in removal rate was not significant with increasing dosage. Secondly, the adsorption capacity showed an inverse trend with the dose, with 574.67 mg g^−1^ at a dose of 5 mg and 152.87 mg g^−1^ at a dose of 25 mg. When the adsorbent dosage was high enough, more adsorption sites could be provided for MB in total; however, many adsorption sites were interdigitated due to the increased agglomeration between the adsorbent particles. It further resulted in many adsorption sites being covered by each other, which, in turn, led to a decrease in the adsorption efficiency of the adsorbent. Thus, by considering the adsorption performance of the adsorbent and the cost of the adsorbent, a suitable adsorbent dose of 10 mg was selected and used for the rest of the experiments.

#### 3.2.2. Effect of pH on Adsorption Performance

During adsorption, the pH of the solution also affected the adsorption effect of the adsorbent. The effect of pH on the adsorption performance of PVA–CA is shown in Figure 5b. As the pH increased from 3.0 to 11.0, the adsorption capacity increased from 110.33 mg g^−1^ to 293.47 mg g^−1^, and the removal rate increased from 36.98% to 97.82%. When the pH of the solution was low, more H^+^ was present in the solution and these H^+^ competed with the cationic MB for the same adsorption sites on PVA–CA, and some of the adsorption sites might be occupied by H^+^. This was not conducive to the adsorption of MB by PVA–CA. In that case, when the pH of the solution was less than 7.0, the H^+^ in the solution would protonate the surface of PVA–CA, which would have an electrostatic repulsive effect on the positively charged MB, thus affecting the adsorption efficiency of PVA–CA on MB. Additionally, the adsorption efficiency of PVA–CA was higher, when the pH of the solution was greater than 7.0. At a higher pH, the surface of PVA–CA deprotonated, and the carboxyl group itself turned into negatively charged anions, i.e., carboxylate ions, which enhanced the electrostatic interaction between anions and MB, and facilitated the adsorption of MB. It should be noted that after the pH exceeded 7.0, the increase in adsorption removal rate tended to flatten, and only increased from 91.44% to 97.82%. Therefore, solutions at pH 7.0 were selected for the rest of the experiments.

#### 3.2.3. Effect of Contact Time on Adsorption Performance

The effect of contact time on the adsorption performance of PVA–CA was investigated at initial concentrations of 50, 100, and 150 mg L^−1^ of MB, respectively (Figure 5c). The adsorption capacity of PVA–CA was at a rapid increase in volume during the first 90 min. The adsorption under 50 mg L^−1^ and 100 mg L^−1^ conditions almost reached equilibrium within 180 min. While the adsorption under 150 mg L^−1^ conditions reached equilibrium at 300 min. The rapid adsorption at the initial stage was mainly caused by a large number of free adsorption sites on the surface of PVA–CA and the high concentration of MB in the solution. Thereafter, as the adsorption proceeded, the number of free adsorption sites on PVA–CA gradually decreased, and the driving force of the MB molecule concentration in the liquid phase also gradually decreased. Thus, the adsorption rate of PVA–CA on MB became slower. In addition, the adsorption capacity of PVA–CA on MB at equilibrium was 94.0, 189.60, and 285.60 mg g^−1^ at solution concentrations of 50, 100, and 150 mg L^−1^, respectively. Thus, the adsorption capacity increased gradually with the increase in the initial concentration of the solution.

#### 3.2.4. Effect of Concentration of MB on Adsorption Performance

To investigate the effect of the initial concentration of MB on the adsorption capacity of PVA–CA, the adsorption capacity of PVA–CA on MB was tested at different MB concentrations at 30, 40, and 50 °C (Figure 5d). The adsorption capacity of PVA–CA increased by increasing the concentration of MB. At 30 °C, the adsorption capacity of PVA–CA was 182.26 mg g^−1^ when the concentration of MB was 100 mg L^−1^, and 457.86 mg g^−1^ when the concentration of MB was 300 mg L^−1^. At a higher concentration of MB, the solution had a larger concentration gradient driving force, which could promote mass transfer between the solid and liquid phases, resulting in an enhanced interaction between MB and PVA–CA. The trend of increasing adsorption capacity slowed down when the concentration of MB exceeded 250 mg L^−1^. The reason could be that as the adsorption proceeded, the adsorption process gradually tended to an equilibrium state. In particular, the adsorption capacity of PVA–CA tended to increase with the increased temperature, indicating that increased temperature was beneficial to the adsorption process.

#### 3.2.5. Effect of Temperature on Adsorption Performance

The diffusion rate of molecules can vary at different temperatures, which in turn can affect the adsorption performance of the adsorbent. As Figure 5e demonstrates, temperature played a significant role in the adsorption performance of PVA–CA at different concentrations of MB. The adsorption capacity of PVA–CA gradually increased with the increasing temperature. At a concentration of MB of 100 mg L^−1^, the adsorption capacity of PVA–CA increased from 182.20 mg g^−1^ to 188.60 mg g^−1^, when the temperature was increased from 30 °C to 50 °C. By increasing the temperature, the adsorption was greatly enhanced, owing to the fact that the temperature-controlled increased migration rate of MB led to an increased chance of contact with the adsorption sites on the surface of PVA–CA. Furthermore, it is also evident from Figure 5e that the adsorption capacity of PVA–CA increased as the concentration of MB increased at the same temperature, suggesting that the concentration of MB affected the adsorption process.

#### 3.2.6. Regeneration of Adsorbents

**Figure 6:**
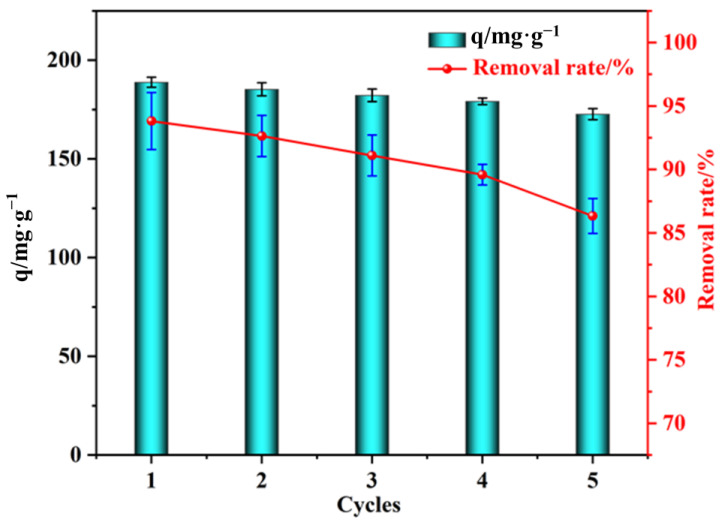
Recycled performance of PVA–CA.

The results of the adsorption–desorption cycle of PVA–CA are shown in Figure 6. The adsorption capacity of PVA–CA gradually decreased with the increase in the number of adsorption–desorption cycles, due to the loss of functional groups on the surface of PVA–CA during each adsorption–desorption cycle. In addition, some of the MB were not completely eluted out and occupied part of the adsorption sites, which eventually led to a gradual decrease in the adsorption capacity of PVA–CA. After five adsorption–desorption cycles, the adsorption capacity of PVA–CA on MB decreased from 188.83 mg g^−1^ to 172.67 mg g^−1^, and the removal rate remained above 85%. This indicates that PVA–CA had good recyclability and potential for practical applications. The prepared PVA–CA was also compared with other literature adsorbents, as shown in Table 1.

### 3.3. Investigation of MB Adsorption Behavior

#### 3.3.1. Adsorption Kinetic Studies

**Figure 7:**
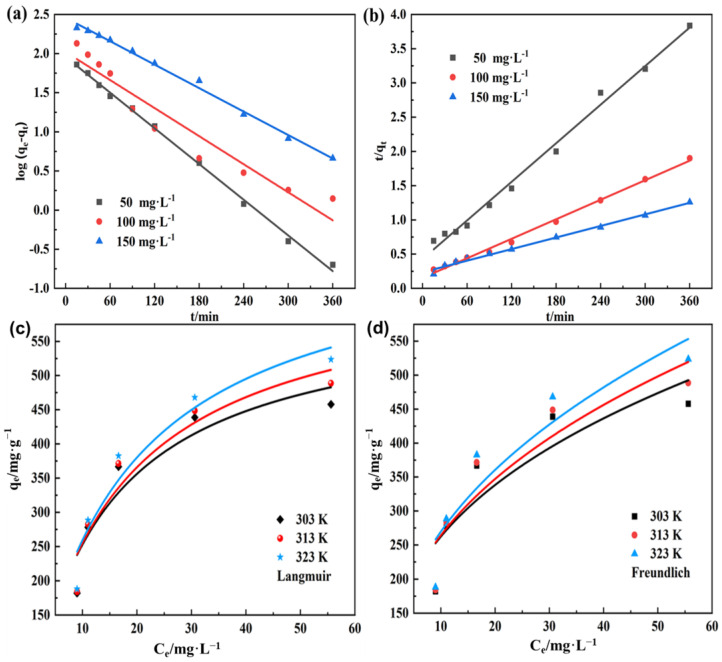
Quasi-first-order (**a**) and quasi-second-order (**b**) kinetic fit curves, Langmuir (**c**) and Freundlich (**d**) adsorption isotherm fitting curves for PVA–CA.

Based on the data obtained in Figure 5c, a linear fit was created using the quasi-first-order and quasi-second-order kinetic models, and the resulting fitted curves are shown in Figure 7a,b, with the relevant kinetic parameters listed in Table 2. The correlation coefficients R^2^ of the quasi-second-order kinetic models fitted at initial concentrations of 50, 100, and 150 mg L^−1^ of MB solution were 0.99145, 0.99566, and 0.99171, respectively, which were higher than those of the corresponding quasi-first-order kinetic models, indicating that the adsorption process was more consistent with the quasi-second-order model. In addition, the theoretical adsorption capacity values obtained from the fitting of the quasi-second-order kinetic model were closer to the experimental values, which further indicates that the quasi-second-order model was more suitable for describing the adsorption process of PVA–CA on MB. The above results suggest that chemisorption may exist in the process of MB adsorption by PVA–CA.

#### 3.3.2. Study of Adsorption Isotherm

To further investigate the adsorption process of MB on PVA–CA, the experimental data were analyzed using the Langmuir and Freundlich adsorption isotherm models, and the fitted curves are shown in Figure 7c,d with the relevant parameters as listed in Table 3. The fitted results show that the correlation coefficients of the Langmuir model at 303, 313, and 323 K were 0.9435, 0.9514, and 0.9728, respectively, which were higher than those of the corresponding Freundlich model. Therefore, the adsorption process of PVA–CA on MB was more consistent with the Langmuir model, indicating that the adsorption behavior tended to be more toward monolayer adsorption. As shown in Table 2, the theoretical maximum adsorption capacities obtained by fitting the Langmuir model were 603.39, 650.72, and 709.86 mg g^−1^ at 303, 313, and 323 K, respectively, indicating that increasing the temperature was conducive to improving the adsorption performance of PVA–CA on MB. In addition, the correlation coefficient R^2^ of the Freundlich model was higher than 0.70, revealing that the model could reflect the adsorption characteristics to some extent, but could not fully describe the adsorption process. The magnitude of the constant 1/n was related to the inhomogeneity of the adsorbent surface and could quantify the ease of the adsorption process. When the value of 1/n was between 0 and 1, the adsorption reaction was favored. The 1/n in the Table 3 are all less than 1, indicating that the adsorption process was relatively easy to carry out under these conditions. In addition, the fitted parameter K_F_ became progressively larger with the increased temperature, indicating that the adsorption process was characterized by more pronounced heat absorption. Therefore, increasing the temperature was favorable for the adsorption process.

#### 3.3.3. Study of Adsorption Thermodynamic Study

**Figure 8:**
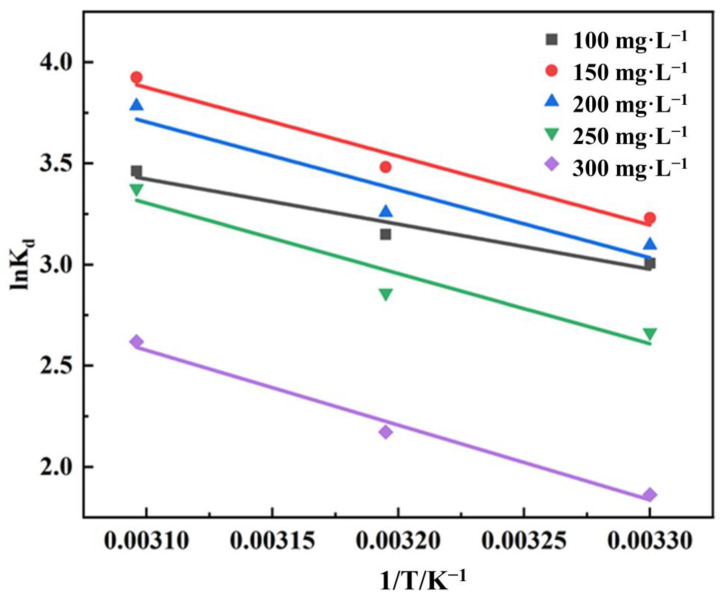
Relationship curves between lnK_d_ and 1/T.

The results of the adsorption thermodynamic fit are shown in Figure 8 and Table 4. ΔG^0^ was negative at all temperatures, indicating that the adsorption of MB by PVA–CA proceeded spontaneously. At concentrations of 100, 150, 200, 250, and 300 mg L^−1^ of MB, ΔH^0^ was 18.5012, 28.2333, 27.8958, 28.9155, and 30.7286 kJ mol^−1^, respectively, all of which were greater than zero, indicating that the adsorption process was heat-absorbing and increased temperature facilitated the adsorption reaction. This was in agreement with the results obtained in Figure 5e. In the case of MB at concentrations of 100, 150, 200, 250, and 300 mg L^−1^, ΔS^0^ was 85.8048, 119.7350, 117.2734, 117.1038, and 116.6739 J mol^−1^ K^−1^, respectively, which were all greater than zero, indicating that the randomness of the solid–liquid interface increased during the adsorption process

### 3.4. Absorption Mechanism

#### 3.4.1. EDS Energy Spectrum Adsorption Validation

**Figure 9:**
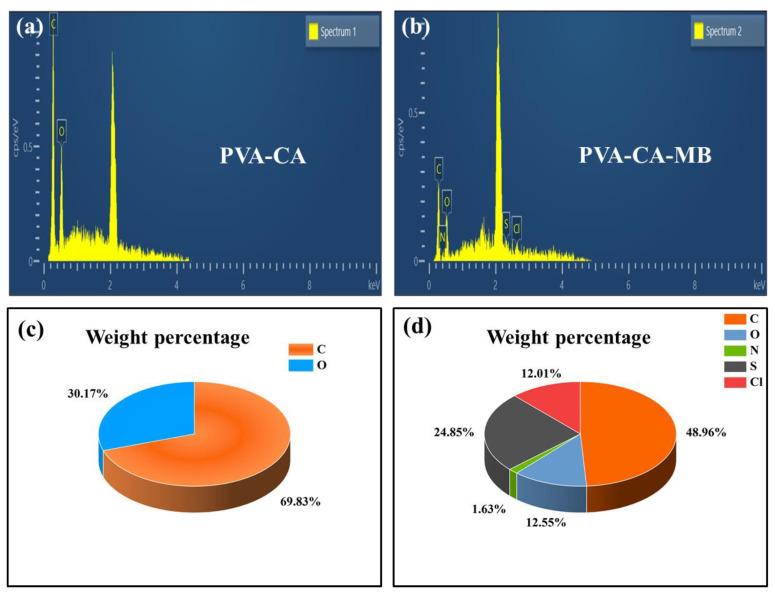
(**a**,**b**) EDS diagrams before and after PVA–CA adsorption; (**c**,**d**) the corresponding elemental weight percentages.

As shown in Figure 9a,c, the elemental distribution of PVA–CA and PVA–CA-MB characterized by EDS confirmed that MB was indeed adsorbed onto PVA–CA. Two elements in PVA–CA were C and O, with weights of C: 69.83% and O: 30.17%, respectively. In contrast, Figure 9b,d show that N, S, and Cl elements were present after the adsorption of MB by PVA–CA, with elemental weights of C: 48.96%, O: 12.55%, N: 1.63%, S: 24.85%, and Cl: 12.01%, respectively. The presence of N, S, and Cl elements confirmed the adsorption of MB onto PVA–CA.

#### 3.4.2. Mechanistic Assumptions

The results of the adsorption experiments show that PVA–CA had good adsorption performance on MB. The adsorption kinetic analysis showed the presence of chemisorption in the adsorption process of PVA–CA on MB. Chemical adsorption generally involves the transfer, exchange, and coexistence of electrons between the adsorbent and the functional groups on the adsorbent surface. Possible mechanisms involved in the adsorption process have been postulated [48]. (1) Electrostatic interactions: the carboxyl groups contained in PVA–CA were readily deprotonated and converted to carboxylate ions under alkaline conditions. The carboxylate ions were bound to the positively charged MB by electrostatic interactions. (2) Hydrogen bonding interactions: the hydroxyl and carboxyl groups contained in PVA–CA were more electronegative and could be hydrogen bonded with MB. (3) Pore adsorption: a pore structure possessed by the adsorbent could provide adsorption sites for MB.

#### 3.4.3. FT-IR Analysis

**Figure 10:**
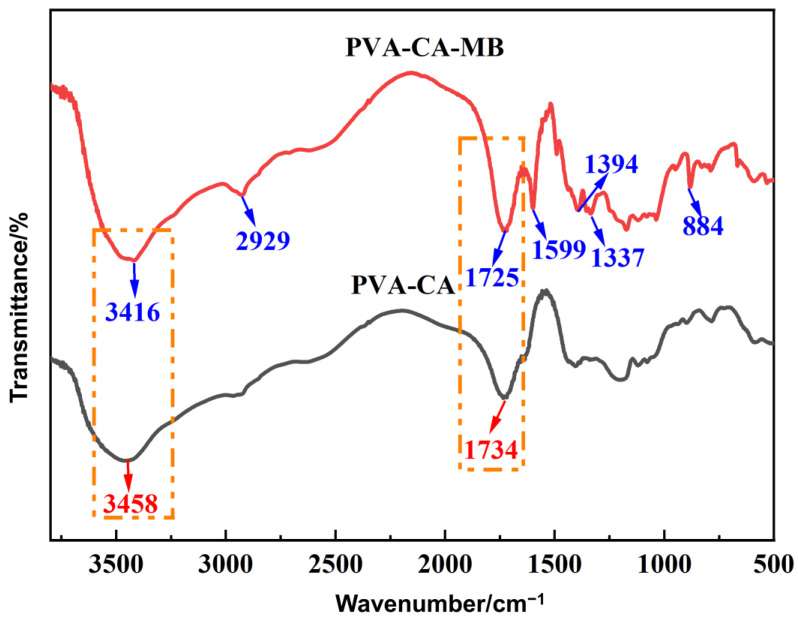
FT-IR spectra of PVA–CA before and after MB adsorption.

In order to further investigate the adsorption mechanism of PVA–CA on MB, the samples before and after adsorption were analyzed by FT-IR spectroscopy, and the results are shown in Figure 10. For the unabsorbed sample PVA–CA, the absorption peak at 3458 cm^−1^ could be attributed to the stretching vibration of the hydroxyl group and the absorption peak at 1734 cm^−1^ could be attributed to the stretching vibration of C=O in the ester group. After the adsorption of MB, new characteristic peaks appeared in the FTIR spectra of PVA–CA-MB, with C=N (1599 cm^−1^) and aromatic C-H (884 cm^−1^) stretching vibration peaks, methyl (1337 cm^−1^) stretching vibration peaks, and C-N (1394 cm^−1^) stretching vibration peaks; the appearance of these characteristic peaks indicates that MB was successfully adsorbed onto PVA–CA. In addition, the peak intensity of the hydroxyl group at 3458 cm^−1^ was significantly weakened and blue-shifted, indicating that there might be hydrogen bonding interactions between the hydroxyl group and MB in PVA–CA. Meanwhile, the characteristic peak of C=O at 1734 cm^−1^ also showed a blue shift after the adsorption of MB, which may be due to the electrostatic interaction between the carboxyl group in PVA–CA and MB. In summary, the adsorption process of MB by PVA–CA may be dominated by chemisorption.

#### 3.4.4. Simulation and Computational Analysis

DFT theoretical simulations were used to further understand the adsorption mechanism of PVA–CA on MB. The adsorption energy was first calculated and the results are shown in Table 5. The total energy of PVA–CA-MB was −4474.5128 Ha, the energy of PVA–CA was −3291.8174 Ha, and the energy of MB was −1182.6135 Ha. The calculated adsorption energy of PVA–CA on MB was −215.0310 kJ mol^−1^.

**Figure 11:**
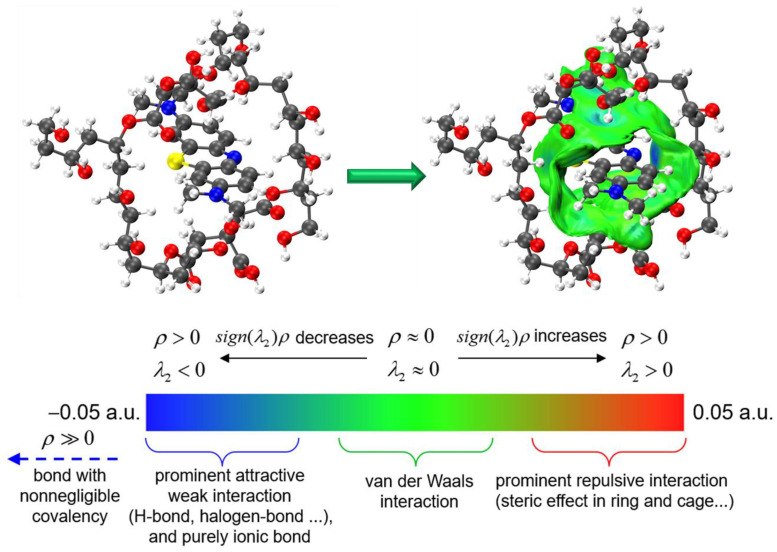
IGMH analysis of the intermolecular interaction between PVA–CA and MB.

Then, IGMH analysis was carried out to present the interactions between PVA–CA and MB, as shown in Figure 11. The blue part is the region of strong attraction, generally including hydrogen bonding, halogen bonding, or other strongly attractive electrostatic interactions, while the green part is the interaction of general strength, such as van der Waals interactions. The interaction regions were mostly green, and it can be assumed that there were strong van der Waals interactions between PVA–CA and MB. Additionally, a few regions showed a blue color, which can be attributed to the hydrogen bonding interaction between N–H–C in MB and the hydroxyl group in PVA–CA, as well as the electrostatic interaction with the carboxyl group.

### 3.5. Generality Testing

**Figure 12:**
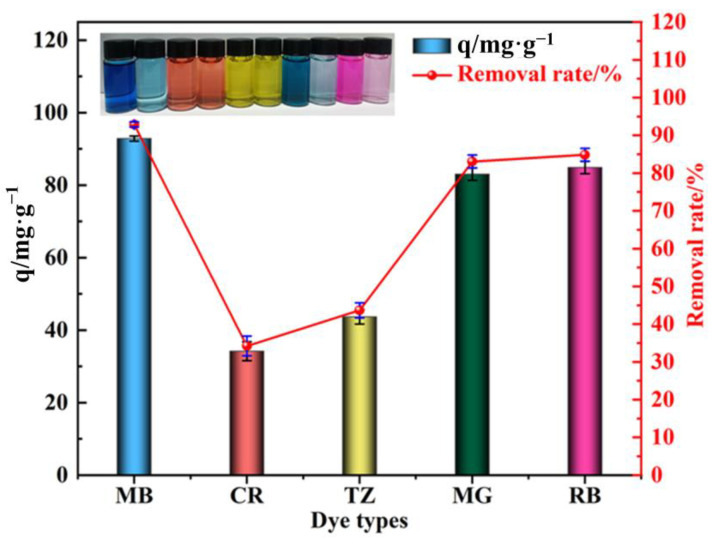
Experimental results of the PVA–CA generality test and comparison of the adsorption capacity of different dyes.

The adsorption performance of PVA–CA on different types of dyes was investigated by selecting MB, CR, MG, TZ, and RB as the target dyes. The specific procedure of the adsorption experiment is presented in the Supporting Information. Figure 12 indicates that PVA–CA had a good adsorption performance for the cationic dyes MB, MG, and RB with removal rates of 92.87, 83.07, and 84.87%, respectively. However, the adsorption performance for the anionic dyes CR and TZ was poor, with removal rates of only 34.20% and 43.67%, respectively. The above results indicate that PVA–CA had a good adsorption effect on cationic dyes. This may be because cationic dyes were positively charged, while the carboxyl group in PVA–CA could be easily ionized into carboxylate ions and negatively charged under aqueous conditions, especially under alkaline conditions, which could generate strong electrostatic interactions between the two components and was beneficial for the adsorption process.

## 4. Conclusions

The composite adsorbent PVA–CA was prepared by esterification, and the optimum preparation conditions of the adsorbent were investigated using single-factor and orthogonal experiments. The adsorption performance, adsorption behavior, and adsorption mechanism of PVA–CA on MB were systematically investigated. The adsorption capacity of PVA–CA on different types of dyes was also evaluated using generality experiments. The adsorption process of PVA–CA on MB was characterized by chemisorption and monolayer adsorption and was spontaneous and heat-absorbing, with increased disorder at the solid–liquid phase interface. The different factors affecting the adsorption performance were further investigated, with the largest adsorption capacity being 709.86 mg g^−1^. The PVA–CA material showed a better adsorption performance towards cationic dyes due to hydrogen bonding interactions and electrostatic interactions. Thus, PVA–CA is a good candidate for the adsorption of cationic dyestuffs in wastewater treatment and has prospective applications in the future.

## Figures and Tables

**Table 1:** Comparison of PVA–CA with other types of adsorbents.

Adsorbent Material	Adsorption Capacity (mg g^−1^)	Ref.
Walnut shell	11.76	[29]
Date pit-activated carbon	39.68	[29]
Polymeric hydrogel	355.9	[40]
Reduced graphene oxide	37.78	[41]
Nickel oxide nanoparticles	93.46	[42]
β-CD conjugated graphene oxide	323.98	[43]
Mg-Silicate	408.0	[44]
Chitosan coated sodium zeolites	287.0	[45]
Cellulose nanofibrils	14.71	[46]
TS–COF–2	445.0	[47]
PVA–CA	709.86	This work

**Table 2:** Relevant parameters of the two kinetic models.

MB Concentration/mg L^−1^		Kinetic Model Categories and Related Parameters					
	q_e_ /mg g^−1^	Quasi-First-Order Kinetic Model			Quasi-Second-Order Kinetic Model		
		q_e,cal_/mg g^−1^	K_1_/min^−1^	R^2^	q_e,cal_/mg g^−1^	K_2_/min^−1^	R^2^
50	94.0	106.49	0.0175	0.97709	90.56	0.00028	0.99145
100	189.6	104.64	0.0137	0.92136	195.53	0.00017	0.99566
150	285.6	354.61	0.0115	0.96437	286.97	0.00003	0.99171

**Table 3:** Parameters associated with the two sorption isotherm models.

Temperature/K	Isothermal Adsorption Model Categories and Related Parameters					
	Langmuir			Freundlich		
	q_max_/mg g^−1^	K_L_ L mg^−1^	R^2^	1/n	K_F_/L mg^−1^	R^2^
303	603.29	0.05767	0.9435	0.4188	102.8605	0.7323
313	650.72	0.06423	0.9514	0.3940	106.6763	0.7901
323	709.86	0.07213	0.9728	0.3670	112.7007	0.8244

**Table 4:** Thermodynamically relevant parameters.

The Concentration of MB/mg L^−1^	Temperature/K	ΔG^0^/kJ mol^−1^	ΔH^0^/kJ mol^−1^	ΔS^0^/J mol^−1^ K^−1^
100	303	−7.4976	18.5012	85.8045
	313	−8.3556		
	323	−9.2137		
150	303	−8.0464	28.2333	119.7350
	313	−9.2438		
	323	−10.4411		
200	303	−7.6380	27.8958	117.2734
	313	−8.8108		
	323	−9.9835		
250	303	−6.5670	28.9155	117.1038
	313	−7.7380		
	323	−8.9090		
300	303	−4.5836	30.7286	116.6739
	313	−5.7503		
	323	−6.9171		

**Table 5:** Adsorption energy of PVA–CA on MB calculated by first principles.

PVA–CA-MB Total Energy/Ha	PVA–CA Energy/Ha	MB Energy/Ha	Adsorption Energy/Ha	Adsorption Energy/kJ mol^−1^
−4474.5128	−3291.8174	−1182.6135	−0.0819	−215.0310

## Data Availability

The data presented in this study are available upon request from the corresponding author.

## Supplementary Materials

The following supporting information can be downloaded at: https://www.mdpi.com/article/10.3390/polym15224341/s1. Figure S1: Procedure diagram for the preparation of PVA-CA. Figure S2: The effect of mass ratio of CA to PVA on the adsorption performance of PVA-CA. Figure S3: The effect of reaction temperature on the adsorption performance of PVA-CA. Figure S4: The effect of reaction time on the adsorption performance of PVA-CA. Figure S5: The influence of the amount of KH_2_PO_4_ on the adsorption performance of PVA-CA. Figure S6: The standard curve of MB solution. Figure S7: Standard curve of each dye solution. Table S1: Orthogonal experiment level and factor table. Table S2: The results of the orthogonal experiments.
