# Supplementary material for: Polyvinyl Alcohol–Citric Acid: A New Material for Green and Efficient Removal of Cationic Dye Wastewater
Source: Polymers (Basel). 2023 Nov 7;15(22):4341. doi: 10.3390/polym15224341 (PMC10675270; doi:10.3390/polym15224341)
Supplement: Supplementary file 1 [file polymers-15-04341-s001.zip › polymers-2691861-supplementary.pdf]

Supporting Information

Polyvinyl Alcohol−Citric Acid: A New Material for Green and Efficient Removal of Cationic Dye Wastewater

Ye He [1,†], Yangyang Zheng [1,†], Xia Liu [2,†], Chang Liu [1], Huacheng Zhang [1,*] and Jie Han [3,*]

[1] School of Chemical Engineering and Technology, Xi'an Jiaotong University, Xi'an 710049, China
[2] Shandong Nonmetallic Materials Institute, Jinan 250031, China
[3] Key Laboratory of Advanced Energy Materials Chemistry (Ministry of Education), College of Chemistry, Nankai University, Tianjin 300071, China
* Correspondence: zhanghuacheng@xjtu.edu.cn (H.Z.); hanjie@nankai.edu.cn (J.H.)
† These authors contributed equally to this work.

1. Figures

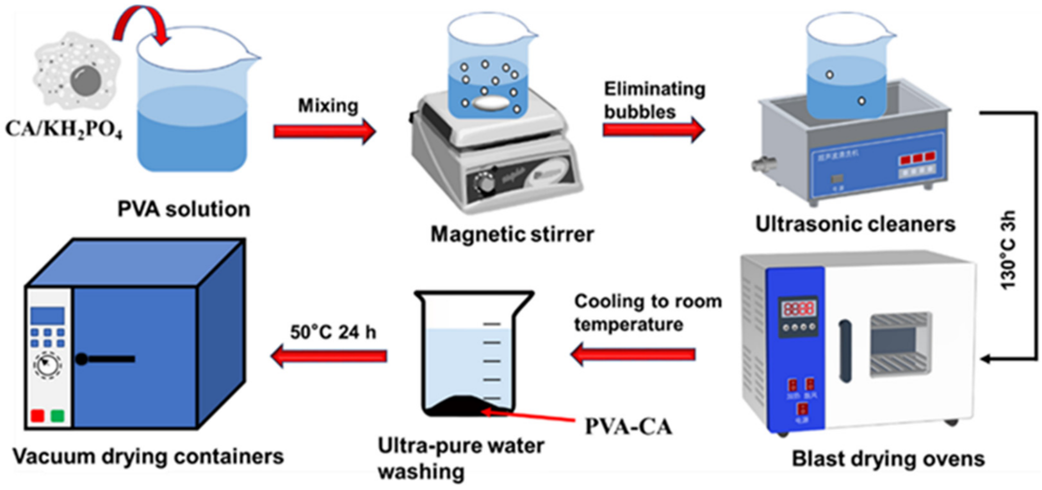

Figure S1 Procedure diagram for the preparation of PVA-CA.

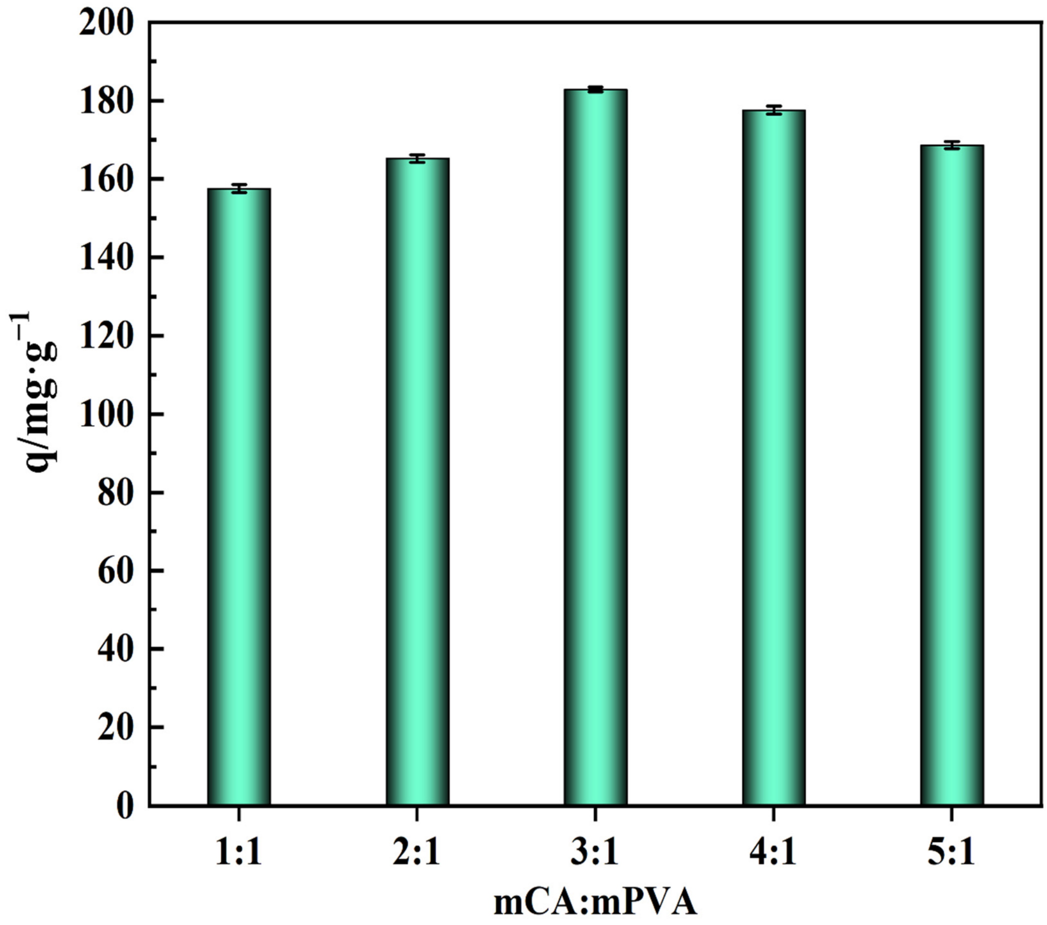

Figure S2 The effect of mass ratio of CA to PVA on the adsorption performance of

PVA-CA, the adsorption capacity of PVA-CA on MB increased with the increase of the mass ratio of CA to PVA, reaching a maximum value of 182.8 mg g^{-1} at a mass ratio of 3/1.

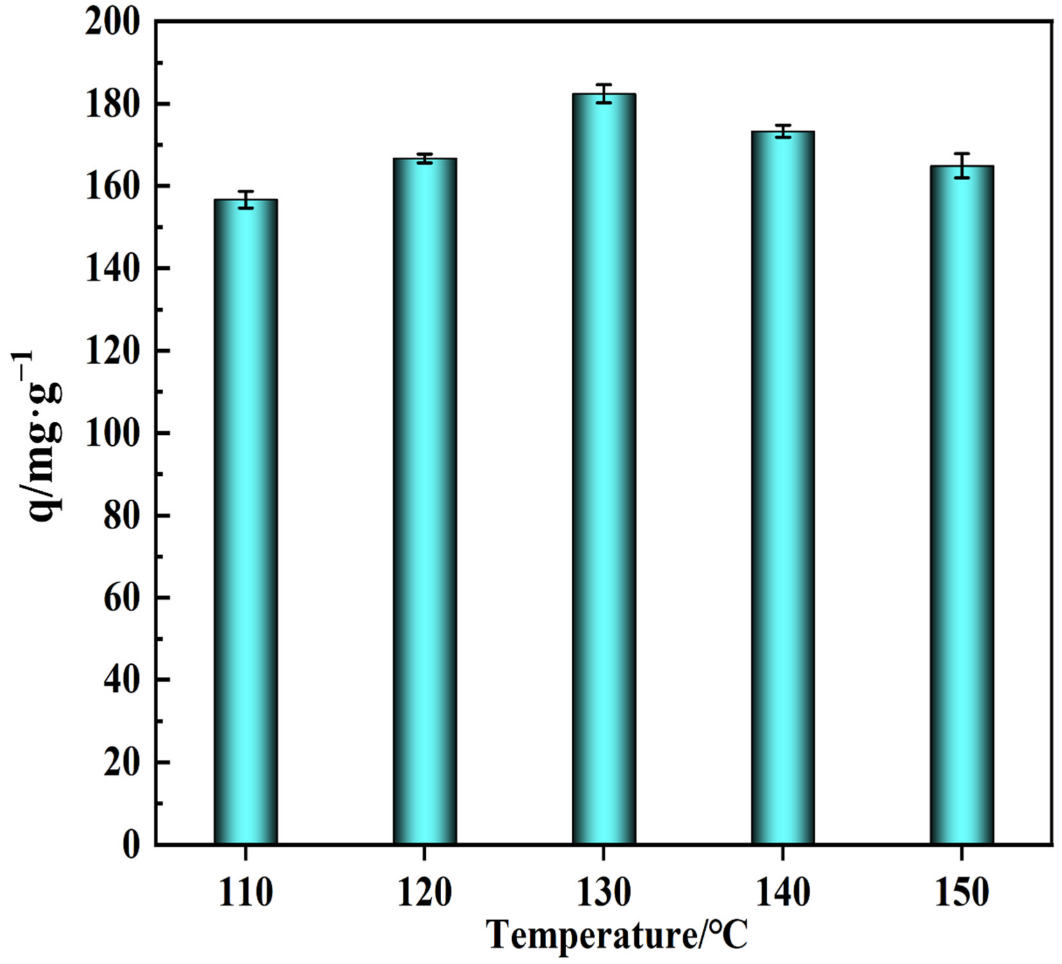

Figure S3 The effect of reaction temperature on the adsorption performance of PVA-CA showed the maximum adsorption capacity of 182.37 mg g^{-1} when the temperature reached 130 °C.

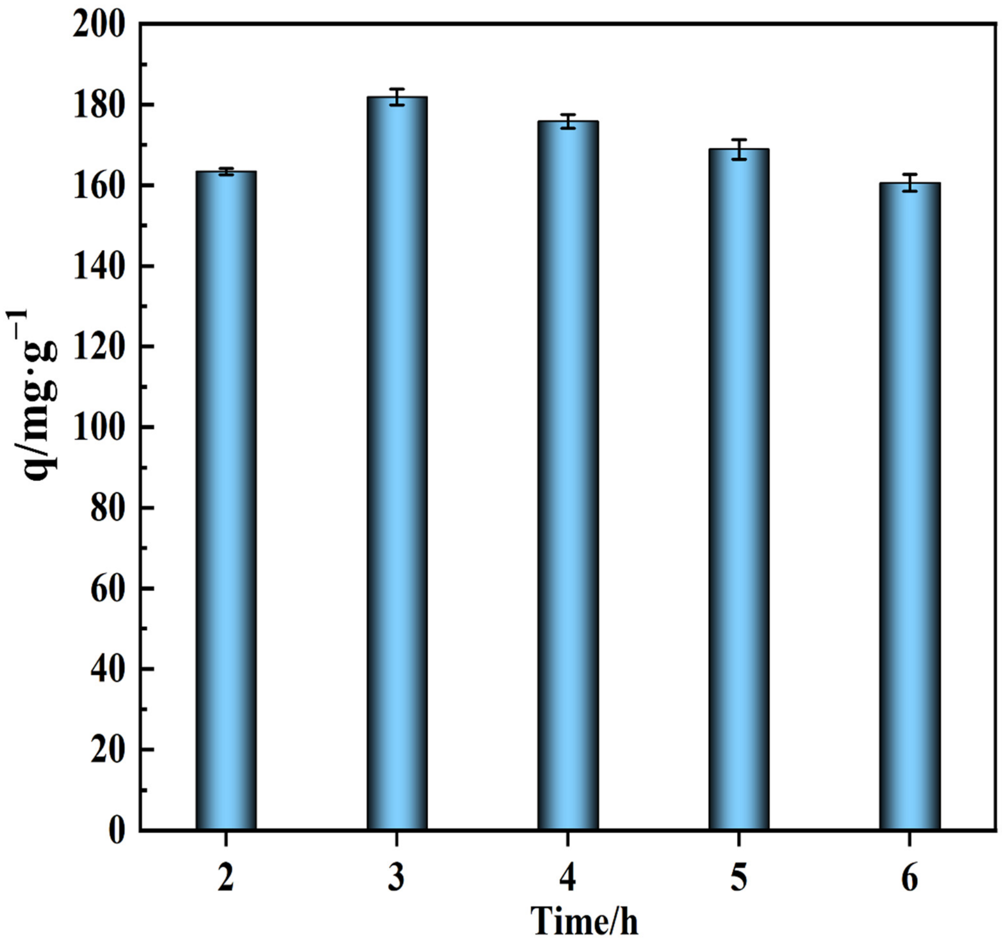

Figure S4 The effect of reaction time on the adsorption performance of PVA-CA, the adsorption capacity of PVA-CA on MB showed a trend of increasing and then decreasing with the increase of reaction time, and had the highest adsorption capacity of 181.87 mg g^{-1} at the reaction time of 3 h.

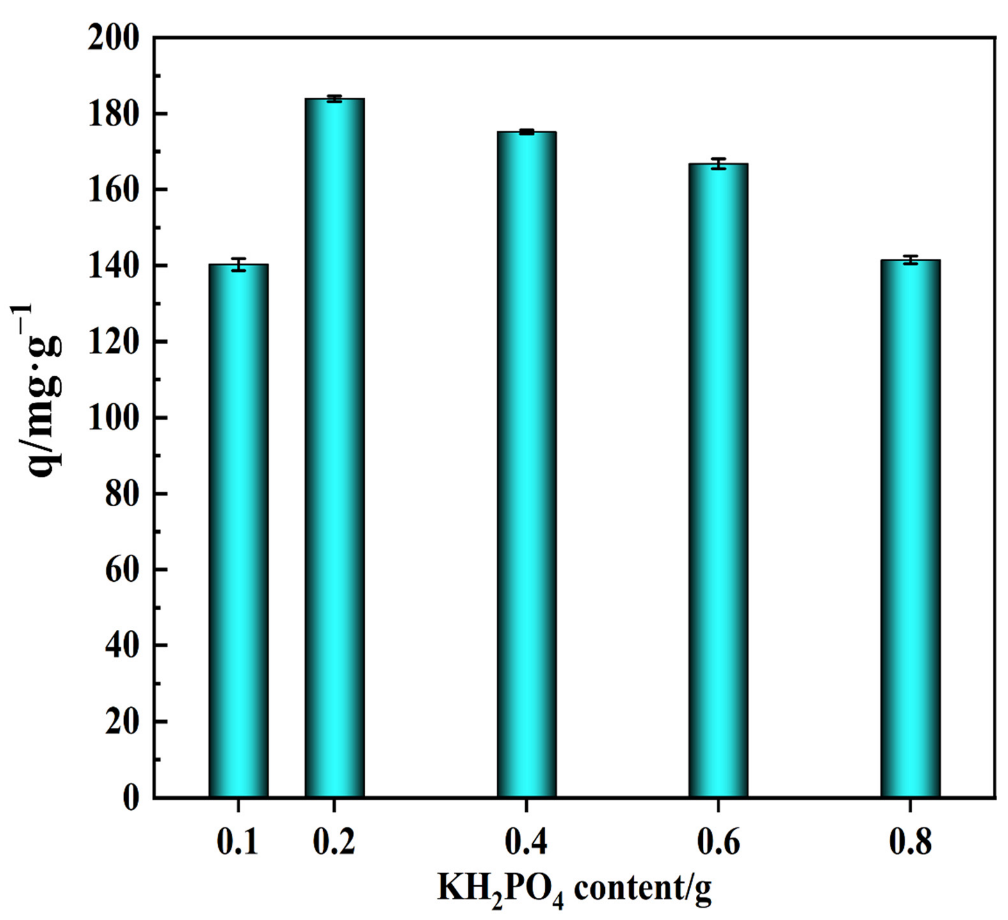

Figure S5 The influence of the amount of KH_2PO_4 on the adsorption performance of PVA-CA, the adsorption capacity of PVA-CA on MB showed a trend of increasing and then decreasing with the increase of the amount of KH_2PO_4. And had the highest adsorption capacity of 183.87 mg g^{-1} at the addition of 0.2 g of KH_2PO_4.

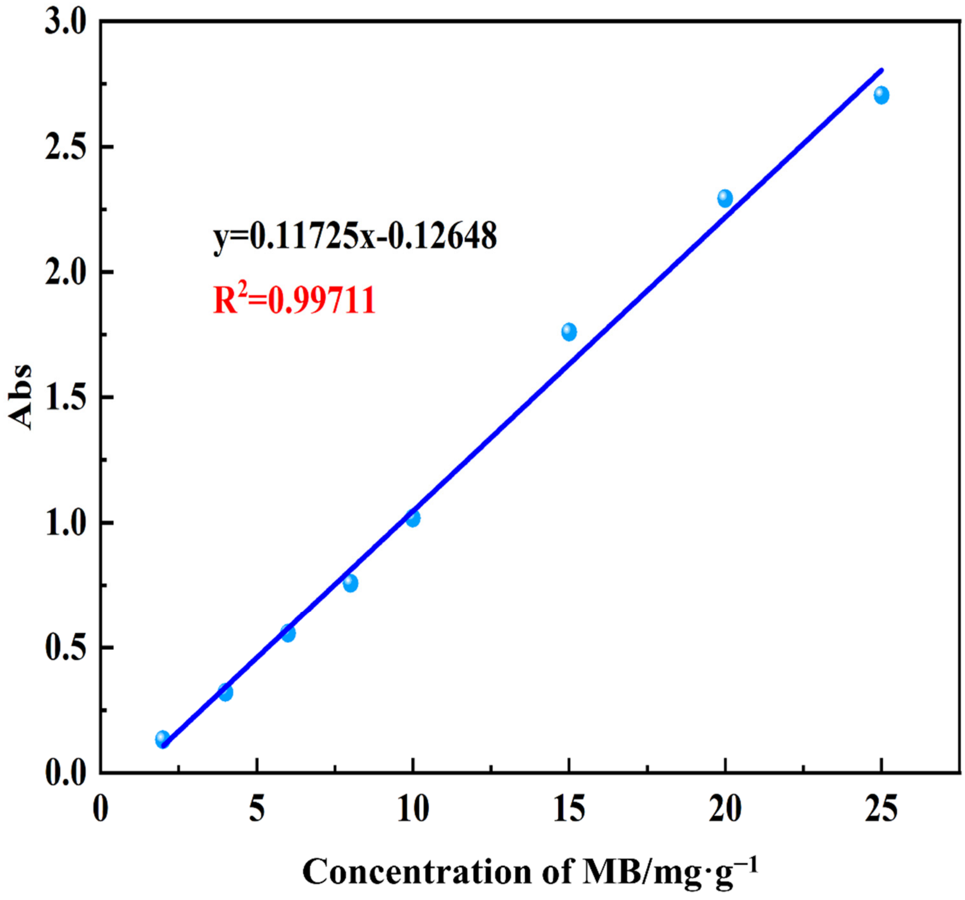

Figure S6 The standard curve of MB solution.

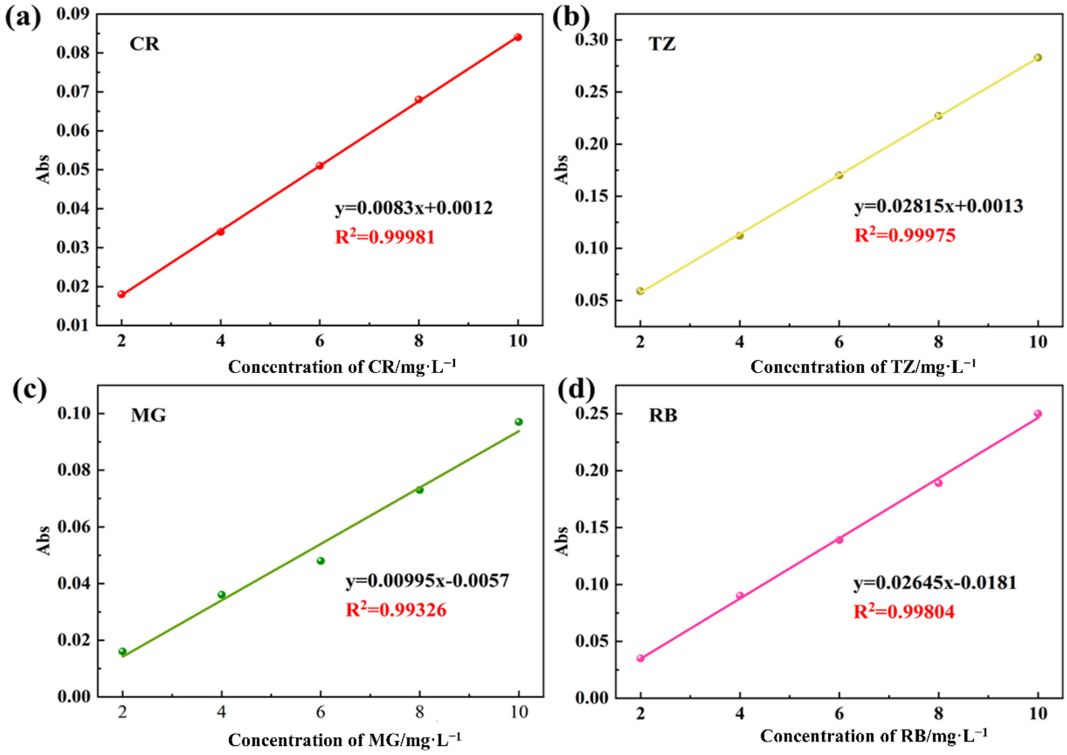

Figure S7 Standard curve of each dye solution. A series of MB solutions were prepared with a concentration range of 0 to 25.0 mg L^{-1} (0, 2.0, 4.0, 6.0, 8.0, 10.0, 15, 20, 25 mg L^{-1}). The absorbance at the maximum absorption wavelength (664 nm) was measured, and the standard curve for the MB was obtained using the MB concentration (C, mg L^{-1}) as the x-coordinate and the absorbance (A) as the y-coordinate (Fig. S6). The experimental results showed a good linear relationship between absorbance and MB concentration with a linear equation of A = -0.12648+0.11725 C (R^2 = 0.99711).

2. Tables

1）Orthogonal experimental design

A three-factor, three-level orthogonal experiment was conducted to explore the best preparation scheme based on a single-factor experiment with the levels and factors shown in Table. S1.

Table. S1 Orthogonal experiment level and factor table.

level	Factors		
	mCA:mPVA<br>A	Temperature/°C<br>B	Time/h<br>C
1	2:1	130	3
2	3:1	140	4
3	4:1	150	5

2）Analysis of orthogonal experimental results

Table. S2 The results of the orthogonal experiments. K_1 denotes the index examined in the experiment where the first level of factors A, B, and C, *i.e.*, mCA:mPVA. K_2 denotes the index examined in the experiment where the second level of factors A, B, and C, *i.e.*, reaction temperature, and K_3 denotes the index examined in the experiment where the third level of factors A, B, and C, *i.e.*, reaction time. The k_1, k_2, and k_3 denote the mean values of K_1, K_2, and K_3, respectively. The r is the extreme difference, which is calculated from the difference between the maximum and minimum values of k_1, k_2, and k_3 in each column. The best solution for this experiment is $A_2B_1C_1$, *i.e.*, the mass ratio of CA and PVA is 3:1, the reaction temperature is 130 °C, and the reaction time is 3 h. This result also agrees with the results of the single-factor experiment.

Number	Factors			
	mCA:mPVA<br>A	Temperature /°C<br>B	Time/h<br>C	Adsorption capacity/mg g^{-1}
1	2:1	130	3	179.6
2	2:1	140	4	162.8
3	2:1	150	5	146.2
4	3:1	130	5	183.2
5	3:1	140	4	181.6
6	3:1	150	3	177.0
7	4:1	130	4	173.6

8	4:1	140	5	184.2
9	4:1	150	3	176.0
K1	488.6	536.4	532.62	
K2	541.8	528.6	517.6	
K3	533.82	499.22	513.6	
k1	162.87	178.8	177.54	
k2	180.6	176.2	172.53	
k3	177.94	166.41	171.2	
R	17.73	12.39	6.34	
